# Birth preparedness and complication readiness (BPCR) among pregnant women in hard-to-reach areas in Bangladesh

**DOI:** 10.1371/journal.pone.0189365

**Published:** 2017-12-11

**Authors:** Md Moinuddin, Aliki Christou, Dewan Md Emdadul Hoque, Tazeen Tahsina, Shumona Sharmin Salam, Sk Masum Billah, Lianne Kuppens, Md Ziaul Matin, Shams El Arifeen

**Affiliations:** 1 Maternal and Child Health Division, icddr,b, Dhaka, Bangladesh; 2 Health Section, UNICEF, Dhaka, Bangladesh; Public Library of Science, FRANCE

## Abstract

**Background:**

Birth preparedness and complication readiness aims to reduce delays in care seeking, promote skilled birth attendance, and facility deliveries. Little is known about birth preparedness practices among populations living in hard-to-reach areas in Bangladesh.

**Objectives:**

To describe levels of birth preparedness and complication readiness among recently delivered women, identify determinants of being better prepared for birth, and assess the impact of greater birth preparedness on maternal and neonatal health practices.

**Methods:**

A cross-sectional survey with 2,897 recently delivered women was undertaken in 2012 as part of an evaluation trial done in five hard-to-reach districts in rural Bangladesh. Mothers were considered well prepared for birth if they adopted two or more of the four birth preparedness components. Descriptive statistics and multivariable logistic regression were used for analysis.

**Results:**

Less than a quarter (24.5%) of women were considered well prepared for birth. Predictors of being well-prepared included: husband’s education (OR = 1.3; CI: 1.1–1.7), district of residence, exposure to media in the form of reading a newspaper (OR = 2.2; CI: 1.2–3.9), receiving home visit by a health worker during pregnancy (OR = 1.5; CI: 1.2–1.8), and receiving at least 3 antenatal care visits from a qualified provider (OR = 1.4; CI: 1.0–1.9). Well-prepared women were more likely to deliver at a health facility (OR = 2.4; CI: 1.9–3.1), use a skilled birth attendant (OR = 2.4, CI: 1.9–3.1), practice clean cord care (OR = 1.3, CI: 1.0–1.5), receive post-natal care from a trained provider within two days of birth for themselves (OR = 2.6, CI: 2.0–3.2) or their newborn (OR = 2.6, CI: 2.1–3.3), and seek care for delivery complications (OR = 1.8, CI: 1.3–2.6).

**Conclusion:**

Greater emphasis on BPCR interventions tailored for hard to reach areas is needed to improve skilled birth attendance, care seeking for complications and essential newborn care and facilitate reductions in maternal and neonatal mortality in low performing districts in Bangladesh.

## Introduction

Each day, for thousands of women and their families, the event of childbirth becomes a reason of unnecessary suffering due to acute obstetric complications and maternal deaths [1, 2]. In 2015 there were an estimated 303,000 maternal deaths globally, the majority of which occurred in sub-Saharan Africa (66%) followed by South Asia (22%)[3]. Bangladesh is one of ten countries that accounts for nearly 60% of the global burden of maternal mortality [4]. Although the maternal mortality ratio (MMR) in Bangladesh declined by 40% from 322 per 100,000 live births in 2001 to 176 per 100,00 live births in 2015 almost achieving the target for MDG 5, the burden of direct obstetric care deaths in the country remains high, comprising almost two-thirds of maternal deaths [4, 5].

To meet the recently agreed upon Sustainable Development Goal (SDG) of achieving a global MMR of 70 requires that countries attain an annual rate of reduction in maternal mortality of 7.5% per year between 2016 and 2030. For Bangladesh, this requires accelerating reductions from the current annual rate of 5.4% [5]. Most maternal deaths can be prevented by ensuring that every mother is attended by a skilled provider during birth, and that the birth takes place in a health facility where access to emergency obstetric care can be ensured [6]. In 2014, only 42% of women in Bangladesh delivered with a skilled birth attendant and 37% delivered at a health facility [7]. Being adequately prepared for birth and for emergency complications can be life-saving for mothers and their newborns, as it reduces delays associated with care-seeking for obstetric emergencies that contribute to the majority of maternal deaths in low-income settings [1].

Birth Preparedness and Complication Readiness (BPCR) is a key component of safe motherhood programmes and a comprehensive strategy aimed at reducing delays around care-seeking, reaching and receiving care during birth, and promoting skilled care at delivery and in the immediate postnatal period [8–10]. It encourages pregnant women to plan and prepare for birth during the antenatal period in the case that unexpected adverse events arise. In particular, BPCR encourages women and families to identify a birth attendant, place for delivery, and make arrangements for transport and money for every birth [11–13]. BPCR ensures that women will reach care before developing any potential complications during childbirth, thereby preventing both maternal and newborn deaths and contributing to progress towards achieving the Sustainable Development Goals to reduce these deaths [8, 14].

BPCR interventions have been widely used and accepted as a strategy for reducing maternal and newborn deaths in several countries [13, 15]. A recent meta-analysis demonstrated that exposure to BPCR interventions was associated with an 18% reduction in neonatal mortality risk and a 28% reduction of in maternal mortality risk [14]. Several studies have shown the positive impact of birth planning on facilitating use of SBAs and increasing facility deliveries [14, 16–18], however a recent systematic review found that although BPCR interventions can result in improved knowledge in preparation for birth and complication it does not always result in increased use of birth attendants [19]. In Nepal, newborn care practices increased significantly from 19 to 29 percentage points when women were exposed to birth preparedness messages [20]. BPCR also helps improve postnatal care (PNC) practices such as care seeking for newborn illness, clean cutting of the umbilical cord and breastfeeding within the first hour after birth [14].

Despite the low quality of existing evidence demonstrating its effectiveness for increasing skilled birth attendance and facility deliveries, BPCR interventions are recommended by the World Health Organization (WHO) to be included as an essential part of ANC packages for women [21]. The key components recommended for inclusion in BPCR interventions are: deciding on the place of birth, birth attendant, knowing the location of the nearest facility for the birth or if complication arise, preparing funds for expenses and any supplies or materials to take the facility, identifying support person to care for other children, arranging transportation to facility or in case of complications, and identification of blood donor. Generally countries adapt the components of BPCR interventions for their context and not all are included in BPCR packages.

BPCR packages can reduce maternal and child mortality through improvement in knowledge, practices and care seeking behavior, little is known about the current status of BPCR in Bangladesh, especially in low performing hard-to-reach districts which experience much higher rates of maternal mortality compared to national estimates [22]. In Bangladesh, hard-to-reach areas are those geographically very remote regions with difficult terrain that are accessible only by boat or foot. There are no paved roads and they are often prone to severe flooding and are particularly vulnerable to climate change. These include remote hilly, and low-lying areas referred to as Haor and Char areas. A Haor is a wet land ecosystem in the north-east of Bangladesh (approximately 80,000 km2) which physically is a bowl or saucer- shaped shallow depression also known as a back swamp area and floods every monsoon. Chars are vegetated islands within river banks and are also extremely difficult to access and prone to frequent flooding and erosion [23]. The Government of Bangladesh recognises 23 sub-districts in the country as hard-to reach and provides a hardship allowance to government service provider working in these regions. These areas comprise around 20% of the geographical area of Bangladesh and are home to an estimated 29 million people [24]. Health care access and coverage of key interventions is a major challenge in these areas, particularly during the rainy season when certain areas can be under water for half the year [25]. Geographical barriers, poor road conditions and the lack of transportation make it difficult to reach the health facility and contribute to low levels of utilization of skilled care during and after childbirth. Compounding these challenges is also a shortage of health service providers in these regions.

This paper presents the findings on BPCR practices of recently delivered women (RDW) from a paired cluster-randomized controlled trial conducted in 14 sub-districts of five low performing districts of Bangladesh to evaluate a Maternal, Neonatal and Child Survival intervention program. In the analysis presented here, we aimed to assess the magnitude of BPCR related activities, and identify determining factors of better birth preparedness and their effect on maternal and newborn health care practices in hard-to-reach populations in Bangladesh. The findings will inform policy and program makers around designing interventions to improve maternal and neonatal health outcomes in hard-to-reach areas of Bangladesh.

## Methods

### Study design and setting

**Fig 1:**
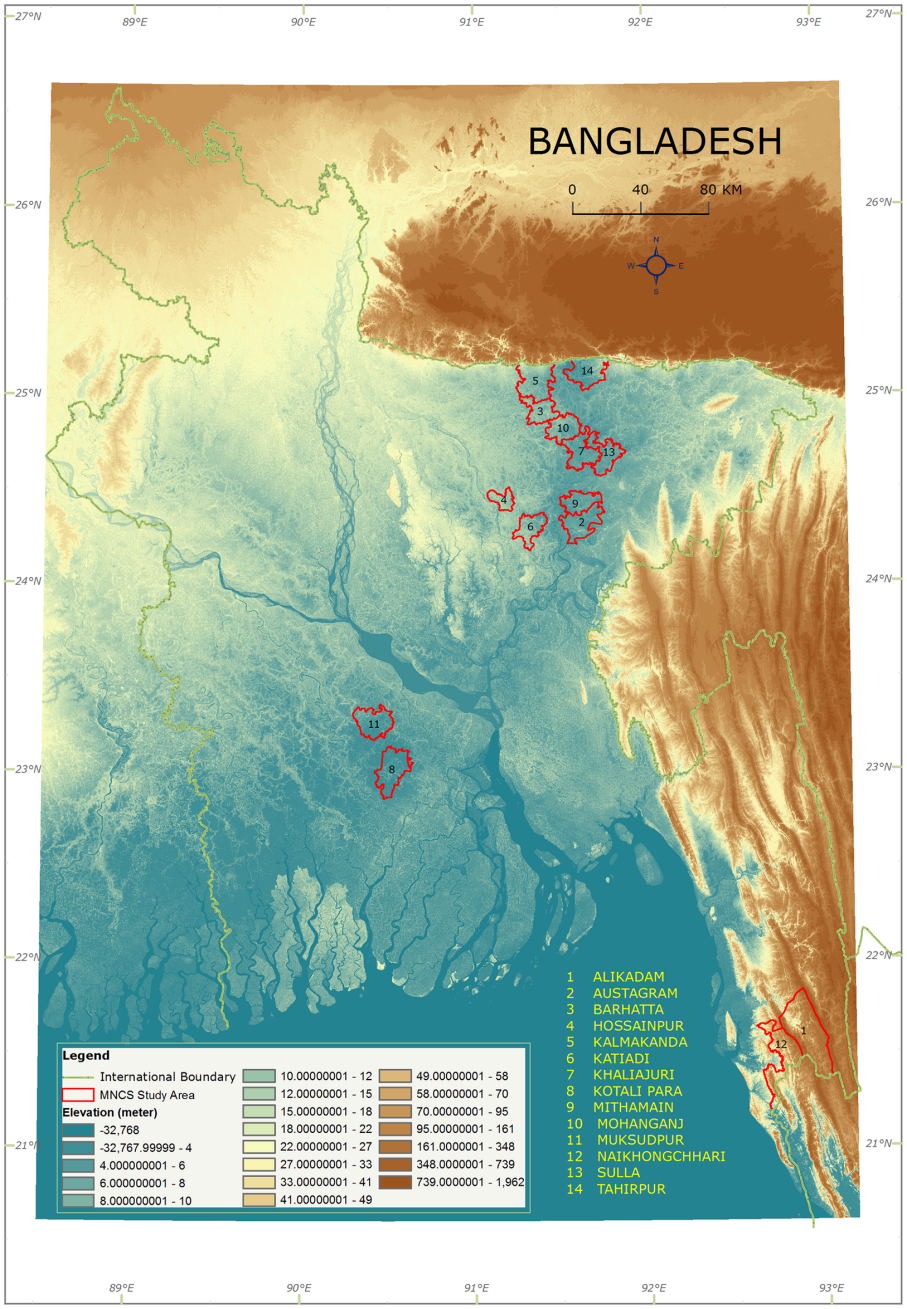
MNCS study sub-districts.

This analysis uses data from the endline survey of a paired cluster-randomized controlled trial carried out in 14 sub-districts of five hard-to-reach districts (Bandarban, Gopalganj, Kishoreganj, Netrokona and Sunamganj) in Bangladesh between 2009 and 2012 (Fig 1). These sub-districts cover an area of approximately 4640 km^2^ with population around 2.5 millions. Each sub-district is characterized by a distinct terrain type;Alikadam and Naikhoncchari sub-district of Bandarban districts are hilly, Sulla and Tahirpur sub-district of Sunamgonj district, Mithamoin, Austogram of Kishorgonj and Khaliajuri of Netrokona districts are haors. Rest of the sub-districts of Sunamganj, Kishoreganj and Netrokona are partially haor. The Muksudpur and Kotalipara of Gopalganj district are riverine plain land with some chars. These sub-districts have very low levels of utilization of health services. In 2010 the percentage of births attended by skilled providers ranged from 10.6% in Sunamganj to 31.4% in Gopalganj, while the proportion of births conducted at health facilities was as low as 9.3% in Sunamganj and was highest in Gopalganj at 23.1% [26]. The aim of the trial was to evaluate an integrated package of Maternal, Neonatal and Child Survival (MNCS) interventions implemented by the Ministry of Health and Family Welfare, Government of Bangladesh (GoB) and UNICEF, in partnership with NGOs, with support from AusAID. The integrated package aimed to accelerate achievements towards meeting MDGs 4 and 5 and included (1) EPI-plus package that promoted immunization, de-worming and distributing vitamin A supplementation; (2) IMCI-plus package focusing on the prevention and treatment of newborn and child illnesses by families, communities and health facilities as well as appropriate feeding practices. (3) ANC-plus package that promoted minimum three antenatal care (ANC) and at least one postnatal care (PNC) from a trained health provider, referral and linkage to the existing GoB and NGO projects such as demand side financing, maternal voucher scheme etc. The channels for reaching the communities included home visits and community case management by community-based GoB/ NGO health workers and community support groups. In addition, local village practitioners (village doctors) were trained to reduce harmful practices and practice appropriate referral. Baseline (2009) and end line (2012) cross-sectional surveys were carried out in all sub-districts of the intervention and comparison arms for the evaluation.

### Sample size and sampling method

A multi-stage sampling scheme was used for both the surveys where 30 villages were selected using Probability Proportion to Size (PPS) sampling method from each sub-district. In the second stage, 31 households were randomly selected from each village to identify the required number of RDW. Structured interviews were conducted with 2,931 women with a recent birth. For the purpose of this analysis, we considered all RDW with a birth outcome in the 15-months preceding the survey, giving a total of sample of 2,897 RDW at end line.

### Data collection and management

A pretested structured questionnaire was administered to all RDW, which included questions on socio-demographic characteristics, reproductive history, and knowledge and practices of maternal and newborn health care. During data collection all questionnaires were checked for consistency and completeness by the field supervisors. All data were coded, and entered into a database.

### Data analysis

BPCR was assessed by asking women if they had completed the following four components during their pregnancy: i) identified a health facility for the delivery, ii) identified a skilled birth attendant, iii) saved money in the case of an emergency, and iv) arranged emergency transport. These arrangements were selected based upon the WHO recommendations for birth and emergency preparedness components excluding blood donation [21, 27]. Identification of a potential blood donor was not included due to unavailability of data.

Women that planned to have their birth at a health facility were considered to have *“identified a health facility”* for their birth. Women were considered as having “identified an SBA” if they had said they planned and identified a skilled birth attendant, or said they planned to have their birth in a health facility. SBAs included all medically trained providers defined according to what is used routinely in the Bangladesh Demographic and Health surveys (DHS) (MBBS doctors, nurses, midwives, paramedics, medical assistant and Community-based Skilled Birth Attendants (CSBA)), though in the last survey report the medical assistant was excluded.

Women were considered “well-prepared” if they reported completing at least two of the four birth preparedness components before delivery. This score was based on the distribution of women with either one, two, three or four BPCR components in our sample; less than 10% of women had completed three or more components with over half completing none, so for this context having completed at least two out of four was considered reasonably well-prepared relative to mothers that had either completed either one or none. Other similar studies have generally considered having at least two or three BPCR components as being well prepared [16, 28, 29].

All available socio-demographic, maternal and antenatal care related variables considered as potential influencing factors on birth preparedness and complication readiness were included in the initial model for analysis. The variables for mother’s age, and educational attainment were categorized from continuous variables for ease of analysis. Husband’s information was collected from currently married women. Due to small numbers in some of the categories for religions other than Muslim, these were grouped and coded as “Other”. For both women and their husbands, employment status was categorized into physical work and professional work. Socioeconomic status was measured at the household level through a composite score constructed using principal component analysis. To develop the score, household level variables such as household possessions, materials used for construction of floor, wall, and roof, drinking water source, toilet facilities, ownership of land and domestic animals were considered.

We used descriptive statistics for describing the socio-demographic, ANC related characteristics, and level of BPCR. The association between birth preparedness and potential influencing factors were assessed using odds ratios generated from binary logistic regression. Multivariable logistic regression with a random intercept was carried out to identify determining factors for well preparedness and adjusted for known confounders. Finally, the effect of well preparedness on maternal and newborn health care practices was analyzed using a logistic regression model after adjusting for known confounders. All data were entered and analyzed using statistical software STATA, version 13, special edition.

### Ethical consideration

Due to low literacy rates in rural communities in Bangladesh and among our primary respondents (care-takers of under-five children which were predominantly married women), it was not possible to obtain written consent. Instead, we obtained verbal informed consent. Before conducting the interview, the interviewer read out the approved consent form in the local language (Bengali) to the respondent and clarified any queries. S/he then sought permission from the respondent about whether they agreed to participate in the study. The interviewer ticked the relevant checkbox on the consent form indicating that the interviewee consented to participate, and then read out the following statement to them, "The information in the consent form was read out loud and the respondent clearly understood the contents of consent form”. The interviewer then signed the form. The Institutional Review Board of ICDDR,B which consists of two independent committees named; Research Review Committee and Ethical Review Committee approved this consent process as part of the approval of the study protocol (PR# 2007–059) in 2008.

## Results

### Socio-demographic and antenatal care characteristics

The socio-demographic and antenatal care characteristics of RDW are presented in Table 1. Over half of RDW were aged over 25 years and had either primary level education or no education. Nearly two thirds of husbands had none or primary level education and nearly three quarters worked in a job doing physical labor. The majority of mothers did not work and less than a quarter had any exposure to media. There was an equal distribution of women represented from each of the wealth quintiles and from the intervention and comparison areas. Slightly more women were sampled from the districts of Netrokona and Kishoreganj compared to the other districts. For about a third of women, the most recent birth was their first child. Only 10% of women had received 3 or more ANC visits. Around a third of women had received a visit by a health worker during their pregnancy.

**Table 1:** Socio-demographic and antenatal care characteristics of recently delivered women, 2012.

Characteristic	Categories	Percentage of women (N = 2,897)
**Mother’s age**	15–24	42.0
	25–49	58.0
**Mother’s education**	No education/primary incomplete	56.0
	Primary complete and higher	44.0
**Religion**	Muslim	81.2
	Others	18.8
**Mother’s occupation**	No paid work	93.6
	Physical work (labor)	3.7
	Non physical work (professional)	2.1
	Other/Don’t know	0.7
**Media exposure**	Read newspaper	2.0
	Listen radio	1.4
	Watch television	19.8
**Husband’s education**	No/primary incomplete	61.9
	Primary complete and higher	37.5
	Don’t know	0.6
**Husband’s occupation**	No work	2.1
	Physical work (labor)	73.4
	Non physical work (professional)	24.6
**Wealth quintile**	Lowest	19.2
	Second	20.5
	Middle	20.8
	Fourth	19.3
	Highest	20.3
**Study area**	Intervention	50.1
	Comparison	49.9
**Administrative district**	Bandarban	13.6
	Netrokona	28.0
	Kishoregonj	27.8
	Sunamgonj	17.9
	Gopalgonj	12.7
**Birth order**	1	28.7
	2–4	56.1
	5+	15.3
**Number of ANC visits received from trained provider**	None or <3 ANC	89.9
	3 or more ANC	10.1
**Source of ANC**	None/ANC from un-trained provider	66.9
	ANC from trained provider	33.1
	Received home visit by health worker^§^ during pregnancy	30.1

^§^Any health worker

### Levels of birth preparedness and complication readiness

Birth preparedness and complication readiness was relatively low among women, particularly for planning for facility delivery (8.1%) and identifying a SBA (12.4%) (Table 2). Saving money was the most frequently reported birth preparedness component undertaken with just under half (46.9%) of women having done so, followed by arranging emergency transport (20.8%). Almost half (47.9%) of women had not taken up any of the four BPCR components, while about a quarter (27.6%) reported taking one preparation. Taking up at least two birth preparedness components was considered as being well prepared; about a quarter (24.5%) of women were considered well-prepared.

**Table 2:** Level of birth preparedness and complication readiness among recently delivered women, 2012.

		Percentage of women (N = 2,897)
**BPCR Component**	Planned facility delivery	8.1
	Identified SBA	12.4
	Saved money	46.9
	Arranged transport	20.8
**Number of preparations taken**	0	47.9
	1	27.6
	2	16.7
	3	4.0
	4	3.8
**Better BPCR**	2 or more	24.5

### Factors associated with women that are well prepared

Unadjusted bivariate analyses (Table 3) showed that women in the intervention area were significantly more likely to be well prepared compared to the comparison area (OR = 1.2; CI: 1.1–1.4). Older women tended to be significantly less well prepared than younger women. Both mother’s and husband’s education level was significantly associated with better birth preparedness as was wealth status but only from the third highest quintile. Non-Muslim women were significantly more likely to be well prepared compared with Muslim mothers (OR = 1.7; CI: 1.4–2.0). Women with husbands working in a professional job were almost two times (OR = 1.8, CI: 0.9–3.4) more likely to be well prepared compared to those who’s husbands did not work or worked in a physical/unskilled job. Having attended at least three ANC visits and receiving ANC from a qualified provider was significantly associated with well-prepared mothers, with both showing that these women were around three times more likely to be well prepared. Furthermore, being visited by a health worker during pregnancy was also significantly related to being well prepared (OR = 1.9; CI = 1.6–2.3). Women whose pregnancy was not their first birth were significantly less likely to be well prepared than women having their first birth. Regional variations in birth preparedness were also noted; women residing in the districts Netrokona, Kishoreganj Sunamganj and Gopalganj were significantly more likely to have higher levels of birth preparedness than women living in Bandarban.

**Table 3:** Socio-demographic and antenatal care factors associated with being well prepared (crude and adjusted odds ratio with 95% CI).

Socio-demographic and ANC related factors	Categories	N	Women well-prepared (%)	COR (95% CI)	AOR (95% CI)
**Study area**	Comparison^1^	1,450	25.9	1.0	1.0
	Intervention	1,447	23.1	1.2^ǂ^(1.0–1.4)	1.0(0.8–1.2)
**Mother’s age**	15–24^1^	1,218	27.9	1.0	1.0
	25–49	1,679	22.0	0.7***(0.6–0.9)	1.0(0.8–1.2)
**Mother’s education**	None/primary incomplete^1^	1,623	18.1	1.0	1.0
	Primary complete or higher	1,274	32.6	2.2*** (1.8–2.6)	1.2(0.9–1.4)
**Birth order**	1^1^	830	30.7	1.0	1.0
	2–4	1,624	23.3	0.7*** (0.6–0.8)	0.8(0.7–1.1)
	5+	443	17.2	0.5*** (0.4–0.6)	0.7(0.5–1.1)
**Mother’s occupation**	No work^1^	2,711	24.5	1.0	
	Physical work (labor)	106	19.8	0.8(0.5–1.2)	
	Non physical work (professional)	60	33.3	1.5(0.9–2.6)	
	Other/don’t know	20	15.0	0.5(0.2–1.9)	
**Religion**	Muslim^1^	2,353	22.6	1.0	1.0
	Others	544	32.7	1.7*** (1.4–2.0)	1.2^ǂ^ (1.0–1.6)
**Husband’s education**	None/primary incomplete ^1^	1,780	18.6	1.0	1.0
	Primary complete or higher	1,077	34.5	2.3*** (1.9–2.7)	1.3**(1.1–1.7)
	Don’t know/missing	18	22.2	1.3(0.4–3.8)	0.9(0.3–2.8)
**Husband’s occupation**	No work^1^	59	22.0	1.0	1.0
	Physical/USK	2,109	21.7	1.0(0.5–1.8)	1.6(0.8–3.1)
	Professional	707	33.5	1.8(0.9–3.4)	1.8^ǂ^ (0.9–3.7)
**Media exposure**	Read newspaper	57	54.4	3.8*** (2.2–6.4)	2.2*(1.2–3.9)
	Listen radio	41	53.7	3.7*** (2.0–6.8)	1.8^ǂ^ (0.9–3.6)
	Watch television	574	36.6	2.1*** (1.7–2.6)	1.2(0.9–1.5)
**Wealth Index**	Lowest^1^	556	15.8	1.0	1.0
	Second	594	18.0	1.2(0.9–1.6)	0.9(0.7–1.3)
	Middle	601	24.5	1.7*** (1.3–2.3)	1.3(0.9–1.7)
	Fourth	558	25.4	1.8*** (1.3–2.4)	1(0.7–1.4)
	Highest	588	38.3	3.3*** (2.5–4.4)	1.3(0.9–1.9)
**Administrative districts**	Bandarban	395	15.7	1.0	1.0
	Netrokona	811	21.2	1.4*(1.1–2.0)	1.6*(1.1–2.3)
	Kishoregonj	804	22.3	1.5**(1.1–2.1)	1.5*(1.0–2.1)
	Sunamgonj	519	27.4	2*** (1.5–2.8)	2.4***(1.7–3.5)
	Gopalgonj	368	41.8	3.9***(2.7–5.4)	2.3***(1.6–3.5)
**Number of ANC received from qualified provider**	<3	2,603	22.1	1.0	1.0
	3 or more	294	45.9	3.0*** (2.3–3.8)	1.4*(1.0–1.9)
**ANC provider**	No/ ANC from un-trained provider^1^	1,939	17.6	1.0	1.0
	ANC from trained^#^ provider	958	38.4	2.9*** (2.5–3.5)	1.9***(1.5–2.3)
**Home visit received by health worker**^§^ **during pregnancy**	No	2,025	20.6	1.0	1.0
	Yes	872	33.5	1.9*** (1.6–2.3)	1.5***(1.2–1.8)

AOR- Adjusted Odds Ratio; ANC- antenatal care; COR- Crude Odd Ratio; CI-confidence Interval; USK-unskilled

^§^Any health worker

^ǂ^ OR is statistically significant with 10% level of significance;

*OR is statistically significant with 5% level of significance;

** OR is statistically significant with 1% level of significance;

*** OR is statistically significant with <1% level of significance

^1^The reference category

^#^ trained provider refers to MBBS doctor, Nurse, Midwife, paramedic, sub-assistant community medical officer (SACMO), and community skilled birth attendant (CSBA)

Table 3 also shows the results of the multiple logistic regression model adjusted for possible confounders. After adjustment, the socio-demographic factors that were found to be independent predictors of being well prepared for birth were: husband’s education with at least primary education or higher (OR = 1.3; CI: 1.1–1.7), exposure to media in form of reading a newspaper (OR = 2.2; CI: 1.2–3.9), and residing in any district apart from Bandabarn district. Maternal care and antenatal care characteristics that were identified as independent predictors of being better prepared for birth included, having been visited by a health worker at home during pregnancy (OR = 1.5; CI: 1.2–1.8), having at least 3 or more ANC visits (OR = 1.4; CI: 1.0–1.9.) and receiving ANC from a qualified provider (OR = 1.9, CI: 1.5–2.3).

### Association between birth preparedness, delivery outcomes, postnatal and newborn care practices

Examining delivery, and birth outcomes, care-seeking practices and essential newborn care practices associated with being well prepared compared to less well prepared for birth showed that well prepared women were significantly more likely to report delivering at a health facility (OR = 2.4; 1.9–3.1) and having the delivery conducted by a skilled birth attendant (OR = 2.4, CI: 1.9–3.1), compared to women with lower levels of birth preparedness (Table 4). Women that were better prepared were significantly more likely to receive PNC visit for themselves (OR = 2.6, CI: 2.0–3.2) or their newborn (OR = 2.6, CI: 2.1–3.3) from a trained provider within two days of birth. Well-prepared women demonstrated slightly higher rates of adoption of essential newborn care (ENC) practices, however, only the practice of clean cord care (OR = 1.3, CI: 1.0–1.5) was significantly higher. When we only considered home deliveries, a similar result was observed with well prepared mothers also showing significant improvements in clean cord care. A significant relationship was evident between women that were well prepared and care seeking for complications from a trained provider for delivery complications (OR = 1.8, CI: 1.3–2.6); however, this was not significant for post-partum complications.

**Table 4:** Birth outcomes, care-seeking behavior, essential newborn care and post-natal care practices according to level of birth preparedness of women.

Maternal and neonatal health care practices	Indicators	Less well- prepared (%)	Well prepared (%)	*Adjusted OR (95% CI)*
	**Number of births (N)**	2,188	709	
**Delivery and outcome**	Facility delivery	8.8	29.1	2.4***(1.9–3.1)
	Stillbirth Rate (per 1000 birth)	23	17	0.8(0.4–1.6)
	Delivered by skilled birth attendant	12.2	35.5	2.4*** (1.9–3.1)
	**Number of live births (N)**	2,137	697	
**Post-natal Care Practices (N = live births)**	Mother received PNC from trained provider^#^ within 2 days of delivery	11.9	36.0	2.6*** (2.0–3.2)
	Newborn received PNC from trained provider^#^ within 2 days of delivery	12.1	36.9	2.6*** (2.1–3.3)
**Essential** **Newborn Care Practices (N = live births)**	Dried within 5 minutes	8.1	11.6	1.2(0.9–1.6)
	Wrapped within 5 minutes	5.8	8.3	1.2(0.8–1.7)
	Dried and wrapped within 5 minutes	4.9	7.6	1.2(0.8–1.8)
	Baby first bathed after 3 days	18.2	25.7	1.1(0.9–1.4)
	Clean cord care	48.2	54.1	1.3*(1.0–1.5)
	Initiated breast-feeding within 1 hr	69.3	69.6	1.0(0.9–1.3)
**Essential Newborn Care Practices (N = live births delivered at home)**	Dried within 5 minutes	8.2	9.4	1.0(0.7–1.5)
	Wrapped within 5 minutes	5.8	6.1	0.9(0.6–1.4)
	Dried and wrapped within 5 minutes	4.9	5.7	1.0(0.6–1.5)
	Baby first bathed after 3 days	15.4	16.9	1(0.8–1.3)
	Clean cord care	50.1	60.6	1.4**(1.1–1.7)
	Initiated breast-feeding within 1 hr	70.3	73.3	1.1(0.9–1.4)
Care-seeking for Complications				
**Care seeking/among those with complication**	Sought care from trained provider for delivery complication	22.5 (N = 714)	44.3 (N = 273)	1.8**(1.3–2.6)
	Sought care from trained provider for post partum complication	17.3 (N = 723)	29.2 (N = 267)	1.0(0.7–1.5)

BPCR- birth preparedness and complication readiness; CI- confidence interval; OR-odds ratio; PNC- postnatal care;

^#^ Trained provider refers to MBBS doctor, Nurse, Midwife, paramedic, sub-assistant community medical officer (SACMO), and community skilled birth attendant (CSBA);

*OR is statistically significant with 5% level of significance

** OR is statistically significant with 1% level of significance

*** OR is statistically significant with <1% level of significance;

## Discussion

Our study findings show that birth preparedness in these five low performing, hard to reach districts in Bangladesh was low. Only a quarter of recently pregnant women were well prepared for birth and had taken at least two measures of BPCR. Coverage of BPCR in our study was similar to that reported for Ethiopia [29], slightly lower than Nepal [18] but substantially lower than several other low-income settings [9, 16, 30]. This may be due to the different definitions applied for determining what constituted being “well prepared” as this varied among the different studies. The fact that our study was situated in very remote areas with difficult terrain may explain the low levels of birth planning observed as many of the sub-districts in our study were located in areas where health facilities were only reachable by boat and/or foot, and where paved roads are limited. Several other studies in the literature from similar settings have also pointed to the influence of remoteness and distance to health facilities, and delivering at health facility [31–33]. The majority of women in our study had planned to have their birth at home—a common practice in Bangladesh particularly in areas where access to a health facility is challenging. Several other factors including poverty, traditional views, restriction on women’s decision making ability, and lack of transportation and fear of caesarean sections, have been identified as important reasons behind the decision for women to deliver at home with untrained birth attendants in Bangladesh [25]. These are important considerations for developing BPCR interventions, particularly for hard to reach areas where reaching a health facility may be impractical, or just not possible and perhaps greater emphasis should be placed on ensuring skilled birth attendance and safe delivery practices.

Of the four BPCR components examined in this study, saving money was the most frequently adopted component. This was followed by arranging transport, with about one in five women making adequate arrangements for transportation to a facility in the case of an emergency. Planning for a facility delivery was very low, as was identifying a SBA. Only 12% of women had identified a SBA for their birth while nationally in Bangladesh, 32% of mothers had used a SBA in 2011 and this recently increased to 42% in 2014 [7, 34], giving some indication of the comparatively lower rates observed in these hard to reach districts.

The only nationally representative data available on birth preparedness in Bangladesh is from the 2010 Bangladesh Maternal Mortality Survey (BMMS) where currently pregnant women were asked about their birth plans. Although not an ideal comparison to our study given that this survey sampled pregnant women as opposed to recently delivered women, it is currently the only national data available. This survey reported that 17% of women in their third trimester had identified a trained provider to assist with delivery, while 50% had identified an untrained provider [5]. The proportion of women reportedly planning to deliver in a facility in our study was only 8%. This is lower than what was found nationally in the 2010 BMMS where 13% of currently pregnant women in their third trimester had discussed or decided to deliver at a health facility [5]. These lower rates of planning for a SBA and facility delivery are indicative of the disparities that exist across the country particularly in hard to reach areas where access to a health facility is a greater challenge for families. Barriers due to distance and availability of transportation are likely to be an issue, however these need to be explored for this specific context. Moreover, given the high proportion of women delivering at home and use of untrained providers such as Traditional Birth Attendants (TBAs), and the low levels of ANC attendance, women may be missing out on important information around birth planning usually delivered during these visits.

In our study, independent predictors of being well-prepared for birth included having a spouse with at least primary education or higher, living in any district apart from Bandarbarn, exposure to media in the form of reading a newspaper, receiving a visit by a health worker at home during pregnancy, and attending at least three ANC visits from trained provider. Mother’s education did not remain a predictor after adjustment, which contrasts findings from several other countries [16, 35]. This is not surprising given women’s lack of autonomy and decision making ability regarding health care seeking in Bangladesh; the 2010 BMMS indicated that decision to seek care for maternal complications outside the home was made by the husband 70% of the time [5]. It is likely that decisions regarding birth planning would be similar. However, women who read the newspaper were more likely to plan for safe delivery, suggesting that some level of literacy is important and that mass media or behavior change communication strategies may be an effective way to encourage birth planning for this context. A study in Uganda also found a significant association between exposure to mass media and birth preparedness [36]. In 2014, approximately 87% of rural households in Bangladesh had access to a mobile phone [7] and given the rapid rise and potential of m-health for communicating health messages, this may be another avenue for delivering BPCR messages that could target husbands as well as mothers [37–39].

Husband’s education was identified as an important factor for better BPCR, similar to findings from Nigeria and Ethiopia [29, 40]. An Ethiopian study found husband’s employment status and those belonging to the upper wealth quintiles were more likely to be well prepared [41]. Such households would be in a better position financially to afford to pay for transportation, supplies and cost of care at the health facility.

The importance of ANC attendance in facilitating birth preparedness was confirmed by our study, and reiterated findings from other studies that show a positive association between the number of ANC visits and improved BPCR practices [9, 16, 29]. Frequent ANC is important for identifying risk factors and counseling women on birth preparedness. Our study found that having three or more ANC visits was a determinant of being well prepared for birth after adjusting for potential confounders. This is in agreement with a study conducted in Tanzania although they assessed four or more ANC visits [35]. In our study, only one in ten women reported receiving at least three ANC visits by a qualified provider, which is substantially lower than the national average for Bangladesh (38% in 2011 and 44% in 2014 for all providers), suggesting that greater efforts need to be made to ensure pregnant women living in these difficult terrains are reached through some means to improve ANC coverage.

BPCR practices were found to be higher among women that reported receiving a home visit by a health worker during their pregnancy. Home-based counseling through CHWs is one of the most effective methods for delivering health messages and improving maternal and neonatal outcomes in rural Bangladesh [42–44], therefore it is not surprising that home visits were a predictor of greater birth preparedness for these remote and hard to reach areas where health facilities may not be as accessible for families—both physically and economically. Ensuring that home visits for pregnant women incorporate birth preparedness messages may be an effective strategy for improving care-seeking and facility deliveries in these hard to reach areas.

Women who were well prepared according to our definition, were more likely to deliver at a health facility, use a SBA, visit a trained provider for a post-natal check-up, and seek care for delivery complications. These findings align with existing literature that support the effectiveness of BPCR in improving rates of facility deliveries and skilled attendance at birth in low-income countries [16–18, 28, 45], and reiterates the importance of birth planning in facilitating greater utilization of health facilities. Women that are prepared for birth are more likely to be aware of the importance of safe delivery and know where to go to seek care.

Well-prepared women in our study were significantly more likely to adhere to specific essential newborn care (ENC) practices including clean cord care. These are critical practices that can reduce neonatal morbidity and mortality and improve overall newborn survival. Pneumonia and sepsis are two major causes of newborn deaths, which arise in part as a result of improper ENC practices including bathing of the newborn for first time before 72 hours of birth, unclean cord cutting and care. A recent meta-analysis found a significant association between BPCR interventions and clean cutting of the umbilical cord, and early initiation of breastfeeding. In addition it showed that BPCR significantly reduced neonatal mortality [14, 20]. Our analysis also showed that specific ENC practices among well-prepared women improved for home deliveries too. This is important for the Bangladesh context where nationally 62% of deliveries are still conducted at home [7] and in the five hard to reach districts where our study was based the rate is even higher, at 89%.

The proportion of women and newborns receiving PNC from trained provider within two days of delivery was almost three times higher among well-prepared women. These practices are likely to have improved as a result of BPCR increasing facility delivery and deliveries by SBAs as newborns delivered at a health facility or by SBAs are more likely to receive appropriate ENC and PNC [46]. Care seeking for delivery complications from a trained provider was significantly higher among well-prepared women. This is a critically important for reducing maternal and neonatal deaths and may have played some part in the lower stillbirth rate observed among women that were better prepared for birth. The level of birth preparedness had no effect on care seeking for post partum complications, which may be a result of the emphasis of BPCR messages on being prepared for the delivery and emergencies during the intrapartum period rather than after birth.

Bangladesh has yet to adopt WHO’s focused antenatal care model which promotes birth planning as a strategy to improving health care seeking behavior to ensure timely and appropriate care during pregnancy, labour, delivery and the postnatal period [47]. As part of Bangladesh’s 2001 Maternal Health Strategy [48] which aimed to reducing maternal mortality and improve maternal health, counseling on birth preparedness and interacting with pregnant women and their families were set as two actions to be addressed during ANC visits in the third trimester. It is not clear to what extent government and NGO ANC providers have implemented this. The 2010 BMMS survey asked pregnant mothers if birth planning was discussed during their ANC visits and found that less than a third of women reported any aspect of birth planning was discussed [5]. This suggests that BPCR messages delivered by ANC providers may be inadequate and further efforts should be made to ensure that every pregnant woman has the opportunity to make a plan for birth during their ANC visits through better monitoring of the content and quality of these visits. Furthermore, what will be critical to considerations for health program and policy makers in developing BPCR interventions for hard-to reach areas of the country is how to deliver these BPCR messages, which components to include, and what modifications may be required to BPCR messages given the geographical challenges, and limitations to accessing health facilities.

Remoteness, difficult terrain, limited transportation and cultural believes are significant challenges in hard to reach areas in terms of ensuring uptake of birth preparedness and in general, adequate levels of healthcare utilization and accessibility for expectant mothers. For these areas tailored and targeted efforts are needed if any improvements are to be observed. A UNDP-led program to improve health services in the hilly region of the Chittagong Hill Tracts region of Bangladesh in 2008 provides a good example of targeted and tailored approach that may be worth adapting or replicating for hilly areas [49]. Moreover, in hilly district anecdotal evidence suggest that if any mother died in the hospital are now allowed to bring back dead body at home which hinders care seeking from facilities. There are 11 different indigenous group in hilly area, so intervention has to design based on their cultural believes and practices. In this program, a strong network of community health workers, mobile satellite clinics and strengthened emergency transport and referral system were introduced to facilitate health care access, and reduce maternal and neonatal deaths. Some of these strategies may have potential to be implemented for several hard to reach areas, particularly seeing as in our study home visitation by a health worker appeared to improved BPCR. As the geographical terrain in the MNCS areas varied from wetland to hilly and Chaor and Haor areas, research and feasibility studies to test what works and what will be acceptable for these different terrains and for the diverse tribal populations residing here is very much needed. The appropriateness of birth preparedness and complication readiness messages being delivered should also be re-examined to identify whether modifications or adaptions are required for hard to reach areas.

The Government of Bangladesh has given priority to accelerating initiatives for improving maternal and child health in low performing and hard to reach areas through the most recent five year national health population and nutrition sector development program for 2017–2022 [50]. This strategy has placed emphasis on trialing alternative models of health care delivery in partnership with NGOs that work in these areas. The development of innovative transportation options and strengthening referral linkages to ensure women can get to a health facility will also be important and can be complemented by the governments’ proposed plans to introduce a new cadre of community workers nationally to raise awareness and encourage use of health services.

### Strengths and limitations of study

The main strength of this study was the large sample of households surveyed from hard to reach areas including both hilly and wetland regions across Bangladesh making the results generalizable to other parts of the country with similar levels of terrain and socio-economic deprivation. The main limitation of this study surrounds recall bias arising from self-reported information collected on BPCR through cross-sectional surveys. In addition, the study did not measure all recommended components of BPCR and was limited to only four components and did not include identifying potential blood donor.

## Conclusions

The results of this study demonstrate that birth preparedness among women in these five hard to reach districts in Bangladesh is low, suggesting that more emphasis on BPCR messages is needed and that BPCR should be integrated into a package of intensive interventions and tailored for areas where difficult terrain and remoteness pose as significant barriers to health care seeking and health facility access. BPCR messages are usually delivered during ANC visits, however only around 10% of women in our study reported receiving at least three ANC visits by a qualified provider, which is substantially lower than the national average. This indicates that greater efforts need to be made to ensure pregnant women living in these difficult terrains are reached through some means to improve ANC coverage, perhaps through more outreach and home visits by government or NGO community health workers which has worked well in the past in Bangladesh [51]. At the same time, the quality of ANC visits particularly around counseling on BPCR messages needs to be improved and better services need to be ensured at community level health facilities. The Bangladesh Demographic and Health Surveys are the main source of nationally representative population health information in the country and currently do not measure coverage of BPCR as part of assessing quality of ANC [34]. Ensuring this information is captured would assist with monitoring and identifying gaps in birth preparedness planning messages delivered through ANC visits.

This is the first study to report on BPCR practices in low-performing districts in Bangladesh, identifying important determining factors underlying the adoption of BPCR components. Here we find that independent predictors of well-prepared women included husband’s education, media exposure, and frequency of ANC visits from trained providers. Having being visited by a health worker was also significant predictor of adopting BPCR practices among the women in this study, thus reinforcing the importance of ensuring BPCR through adequate counseling during home visits. Well-prepared women in our study had higher rates of facility deliveries, use of SBA, and care seeking for delivery complications, supporting the value of birth preparedness interventions in contributing to better outcomes for both mothers and newborns.

## Supporting information

S1 Dataset BPCR individual level dataset. (DTA)

## References

[1] Thaddeus S, Maine D. Too far to walk: maternal mortality in context. Social Science and Medicine. 1994;38(8):1091–110. 8042057 10.1016/0277-9536(94)90226-7

[2] WHO. Preventing maternal deaths. Geneva: World Health Organization, 1989.

[3] Alkema L, Chou D, Hogan D, Zhang S, Moller A-B, Gemmill A, et al Global, regional, and national levels and trends in maternal mortality between 1990 and 2015, with scenario-based projections to 2030: a systematic analysis by the UN Maternal Mortality Estimation Inter-Agency Group. The Lancet. 2015;387(10017):462–74. doi: 10.1016/S0140-6736(15)00838-7 26584737 10.1016/S0140-6736(15)00838-7 PMC5515236

[4] WHO, UNICEF, UNFPA, World Bank Group, United Nations Population Division. Trends in Maternal Mortality: 1990 to 2015. Geneva: World Health Organization, 2015.

[5] National Institute of Population Research and Training, MEASURE Evaluation, icddr b. Bangladesh Maternal Mortality and Healthcare Survey 2010. Dhaka, Bangladesh: NIPORT, MEASURE Evaluation, and icddr,b, 2012.

[6] Campbell OMR, Graham WJ. Strategies for reducing maternal mortality: getting on with what works. The Lancet. 2006;368(9543):1284–99. doi: 10.1016/S0140-6736(06)69381-1 10.1016/S0140-6736(06)69381-1 17027735

[7] National Institute of Population Research and Training (NIPORT), Mitra and Associates, ICF International. Bangladesh Demographic and Health Survey 2014: Key Indicators. Dhaka, Bangladesh, and Rockville, Maryland, USA: NIPORT, Mitra and Associates, and ICF International, 2015.

[8] The White Ribbon Alliance for Safe Motherhood/India. Saving Mothers’ Lives, What Works: A Field Guide for Implementing Best Practices in Safe Motherhood. New Delhi, India: The White Ribbon Alliance for Safe Motherhood, India, Sub-committee BP; 2002.

[9] Agarwal S, Sethi V, Srivastava K, Jha PK, Baqui AH. Birth preparedness and complication readiness among slum women in Indore city, India. Journal of health, population, and nutrition. 2010;28(4):383–91. Epub 2010/09/10. 20824982 10.3329/jhpn.v28i4.6045 PMC2965330

[10] JHPIEGO. Monitoring birth preparedness and complication readiness: tools and indicators for maternal and newborn health. Baltimore, Maryland: Johns Hopkins, Bloomberg school of Public Health, Center for communication programs, Family Care International, 2004.

[11] JHPIEGO. Improving safe motherhood through shared responsibility and collective action: the maternal and neonatal health program, accomplishmetns and results 2002–2003. Baltimore, Maryland, USA: United States Agency for International Development, 2004.

[12] WHO. Integrated management of pregnancy and childbirth: standards for maternal and neonatal care. Geneva: World Health Organization, DepartmentofMakingPregnancySafer; 2007.

[13] WHO. Working with individuals, families and communities to improve maternal and newborn health. Geneva: World Health Organization, 2010.

[14] Soubeiga D, Gauvin L, Hatem MA, Johri M. Birth preparedness and complication readiness (BPCR) interventions to reduce maternal and neonatal mortality in developing countries: systematic review and meta-analysis. BMC pregnancy and childbirth. 2014;14:129 Epub 2014/04/09. doi: 10.1186/1471-2393-14-129 24708719 10.1186/1471-2393-14-129 PMC4234142

[15] Roth DM, Mbizvo MT. Promoting safe motherhood in the community: the case for strategies that include men. African Journal of Reproductive Health. 2001;5(2):10–21. 12471909

[16] Bintabara D, Mohamed MA, Mghamba J, Wasswa P, Mpembeni RN. Birth preparedness and complication readiness among recently delivered women in chamwino district, central Tanzania: a cross sectional study. Reproductive health. 2015;12:44 Epub 2015/05/20. doi: 10.1186/s12978-015-0041-8 25981513 10.1186/s12978-015-0041-8 PMC4447013

[17] Magoma M, Requejo J, Campbell O, Cousens S, Merialdi M, Filippi V. The effectiveness of birth plans in increasing use of skilled care at delivery and postnatal care in rural Tanzania: a cluster randomised trial. Tropical Medicine & International Health. 2013;18(4):435–43. 23383733 10.1111/tmi.12069

[18] Nawal D, Goli S. Birth preparedness and its effect on place of delivery and post-natal check-ups in Nepal. PloS one. 2013;8(5):e60957 Epub 2013/05/22. doi: 10.1371/journal.pone.0060957 23690921 10.1371/journal.pone.0060957 PMC3655026

[19] Solnes Miltenburg A, Roggeveen Y, Shields L, van Elteren M, van Roosmalen J, Stekelenburg J, et al Impact of Birth Preparedness and Complication Readiness Interventions on Birth with a Skilled Attendant: A Systematic Review. PloS one. 2015;10(11):e0143382 doi: 10.1371/journal.pone.0143382 . 26599677 10.1371/journal.pone.0143382 PMC4658103

[20] McPherson RA, Khadka N, Moore JM, Sharma M. Are birth-preparedness programmes effective? Results from a field trial in Siraha district, Nepal. Journal of health, population, and nutrition. 2006;24(4):479–88. Epub 2007/06/27. 17591345 PMC3001152

[21] World Health Organisation. WHO Recommendations on Health Promotion Interventions for Maternal and Newborn Health 2015. Geneva, Switzerland: WHO, 2015. 26180864

[22] Ahmed S, Hill K. Maternal mortality estimation at the subnational level: a model-based method with an application to Bangladesh. Bulletin of the World Health Organization. 2011;89(1):12–21. doi: 10.2471/BLT.10.076851 21346886 10.2471/BLT.10.076851 PMC3040017

[23] Sarker M, Huque I, Alam M, Koudstaal R. Rivers, chars and char dwellers of Bangladesh. International Journal of River Basin Management. 2003;1(1):61–80.

[24] Government of Bangladesh. National Strategy for Water and Sanitation: Hard to Reach Areas of Bangladesh. In: Division LG, editor. Dhaka2011.

[25] Sarker BK, Rahman M, Rahman T, Hossain J, Reichenbach L, Mitra DK. Reasons for Preference of Home Delivery with Traditional Birth Attendants (TBAs) in Rural Bangladesh: A Qualitative Exploration. PloS one. 2016;11(1):e0146161 doi: 10.1371/journal.pone.0146161 26731276 10.1371/journal.pone.0146161 PMC4701391

[26] National Institute of Population Research and Training (NIPORT), MEASURE Evaluation, UNC-CH U, ICDDR B. Bangladesh District Level Socio-demographic and Health Care Utilization Indicators. North Carolina: Measure Evaluation, Caroline Population Centre, University of North Carolina at Chapel Hill, 2011.

[27] World Health Organization Birth and emergency preparedness in antenatal care Standards for Maternal and Neonatal Care. Geneva, Switzerland: WHO; 2002.

[28] Tura G, Afework MF, Yalew AW. The effect of birth preparedness and complication readiness on skilled care use: a prospective follow-up study in Southwest Ethiopia. Reproductive health. 2014;11:60 Epub 2014/08/06. doi: 10.1186/1742-4755-11-60 25091203 10.1186/1742-4755-11-60 PMC4127036

[29] Hailu M, Gebremariam A, Alemseged F, Deribe K. Birth preparedness and complication readiness among pregnant women in Southern Ethiopia. PloS one. 2011;6(6):e21432 Epub 2011/07/07. doi: 10.1371/journal.pone.0021432 21731747 10.1371/journal.pone.0021432 PMC3120869

[30] Kabakyenga JK, Ostergren PO, Turyakira E, Pettersson KO. Influence of birth preparedness, decision-making on location of birth and assistance by skilled birth attendants among women in south-western Uganda. PloS one. 2012;7(4):e35747 Epub 2012/05/05. doi: 10.1371/journal.pone.0035747 22558214 10.1371/journal.pone.0035747 PMC3338788

[31] Lohela TJ, Campbell OMR, Gabrysch S. Distance to care, facility delivery and early neonatal mortality in Malawi and Zambia. PLoS ONE. 2012;7(12):e52110 doi: 10.1371/journal.pone.0052110 23300599 10.1371/journal.pone.0052110 PMC3531405

[32] Gabrysch S, Cousens S, Cox J, Campbell OMR. The Influence of Distance and Level of Care on Delivery Place in Rural Zambia: A Study of Linked National Data in a Geographic Information System. PLOS Medicine. 2011;8(1):e1000394 doi: 10.1371/journal.pmed.1000394 21283606 10.1371/journal.pmed.1000394 PMC3026699

[33] Kruger C, Olsen O, Mighay E, Ali M. Where do women give birth in rural Tanzania? Rural and remote health. 2011;11(1791):1–10. 21875299

[34] National Institute of Population Research and Training (NIPORT), Mitra and Associates, ICF International. Bangladesh Demographic and Health Survey 2011. Dhaka, Bangladesh and Calverton, Maryland, USA: NIPORT, Mitra and Associates, and ICF International, 2013.

[35] Urassa DP, Pembe AB, Mganga F. Birth preparedness and complication readiness among women in Mpwapwa district, Tanzania. Tanzania journal of health research. 2012;14(1):42–7. Epub 2012/01/01. . 26591746 10.4314/thrb.v14i1.8

[36] Asp G, Odberg Pettersson K, Sandberg J, Kabakyenga J, Agardh A. Associations between mass media exposure and birth preparedness among women in southwestern Uganda: a community-based survey. Global health action. 2014;7:22904 Epub 2014/01/18. doi: 10.3402/gha.v7.22904 . 24433945 10.3402/gha.v7.22904 PMC3888909

[37] Sarwar MR. Bangladesh Health Service Delivery: Innovative NGO and Private Sector Partnerships. IDS Bulletin. 2015;46(3):17–28. doi: 10.1111/1759-5436.12141

[38] Sondaal SFV, Browne JL, Amoakoh-Coleman M, Borgstein A, Miltenburg AS, Verwijs M, et al Assessing the Effect of mHealth Interventions in Improving Maternal and Neonatal Care in Low- and Middle-Income Countries: A Systematic Review. PloS one. 2016;11(5):e0154664 doi: 10.1371/journal.pone.0154664 27144393 10.1371/journal.pone.0154664 PMC4856298

[39] Lee SH, Nurmatov UB, Nwaru BI, Mukherjee M, Grant L, Pagliari C. Effectiveness of mHealth interventions for maternal, newborn and child health in low-and middle-income countries: systematic review and meta-analysis. Journal of Global Health. 2016;6(1):010401 doi: 10.7189/jogh.06.010401 26649177 10.7189/jogh.06.010401 PMC4643860

[40] Emma-Ukaegbu UC N H, Uzochukwu B.S.C. An assessment of birth preparedness and complication readiness in antenatal women in Umuahia north local government area, Abia State, Nigeria. IOSR Journal of Dental and Medical Sciences (IOSR-JDMS) 2014; (13 (1):90–94).

[41] Debelew GT, Afework MF, Yalew AW. Factors affecting birth preparedness and complication readiness in Jimma Zone, Southwest Ethiopia: a multilevel analysis. The Pan African medical journal. 2014;19:272 Epub 2014/01/01. doi: 10.11604/pamj.2014.19.272.4244 25870727 10.11604/pamj.2014.19.272.4244 PMC4391899

[42] Baqui AH, El-Arifeen S, Darmstadt GL, Ahmed S, Williams EK, Seraji HR, et al Effect of community-based newborn-care intervention package implemented through two service-delivery strategies in Sylhet district, Bangladesh: a cluster-randomised controlled trial. The Lancet. 2008;371(9628):1936–44. 10.1016/S0140-6736(08)60835-1 18539225

[43] El-Saharty S, Susan Powers Sparkes, Helene Barroy, Karar Zunaid Ahsan, and Syed Masud Ahmed. The path to universal health care in Bangladesh: bridging the gap of human resources for health. Washington D.C: World Bank, 2015.

[44] NGO Forum for Public Health. Being Beside the Hard-to-Reach. Dhaka. Dhaka, Bangladesh: NGO Forum for Public Health, 2013.

[45] Moran AC, Sangli G, Dineen R, Rawlins B, Yameogo M, Baya B. Birth-preparedness for maternal health: findings from Koupela District, Burkina Faso. Journal of health, population, and nutrition. 2006;24(4):489–97. Epub 2007/06/27. 17591346 PMC3001153

[46] Callaghan-Koru JA, Seifu A, Tholandi M, de Graft-Johnson J, Daniel E, Rawlins B, et al Newborn care practices at home and in health facilities in 4 regions of Ethiopia. BMC pediatrics. 2013;13:198 Epub 2013/12/03. doi: 10.1186/1471-2431-13-198 24289501 10.1186/1471-2431-13-198 PMC4219496

[47] August F, Pembe AB, Kayombo E, Mbekenga C, Axemo P, Darj E. Birth preparedness and complication readiness—a qualitative study among community members in rural Tanzania. Global health action. 2015;8:26922 Epub 2015/06/17. doi: 10.3402/gha.v8.26922 26077145 10.3402/gha.v8.26922 PMC4468055

[48] Government of Bangladesh. Bangladesh National Strategy for Maternal Health, Bangladesh. In: Ministry of Health and Family Welfare G, editor. Dhaka, Bangladesh2001.

[49] United Nations Development Program (UNDP) Bangladesh. Improving Health, Nutrition and Population in the Chittagong Hill Tracts (1 December 2008–29 February 2012): Project Completion Report. Dhaka, Bangladesh: UNDP, European Union, 2012.

[50] Ministry of Health and Family Welfare, Government of Bangladesh. Program Implmentation Plan: Health Population and Nutrition Sector Program (HPNSP) 2017–2022. Dhaka, Bangladesh: Ministry of Health and Family Welfare (MOHFW), 2017.

[51] El Arifeen S, Christou A, Reichenbach L, Osman FA, Azad K, Islam KS, et al Community-based approaches and partnerships: innovations in health-service delivery in Bangladesh. Lancet. 2013;382(9909):2012–26. doi: 10.1016/S0140-6736(13)62149-2 . 24268607 10.1016/S0140-6736(13)62149-2

